# Transcriptome Analysis of *Paraburkholderia phymatum* under Nitrogen Starvation and during Symbiosis with *Phaseolus Vulgaris*

**DOI:** 10.3390/genes8120389

**Published:** 2017-12-15

**Authors:** Martina Lardi, Yilei Liu, Gabriela Purtschert, Samanta Bolzan de Campos, Gabriella Pessi

**Affiliations:** Department of Plant and Microbial Biology, University of Zurich, CH-8057 Zurich, Switzerland; martina.lardi@uzh.ch (M.L.); yilei.liu@botinst.uzh.ch (Y.L.); gabriela.p@access.uzh.ch (G.Pu.); samanta.bolzandecampos@botinst.uzh.ch (S.B.d.C.)

**Keywords:** differential gene expression, rhizobia, infection, nitrogen fixation, nodulation, NifA, cytochrome, sigma factor, exopolysaccharides, motility

## Abstract

*Paraburkholderia phymatum* belongs to the β-subclass of proteobacteria. It has recently been shown to be able to nodulate and fix nitrogen in symbiosis with several mimosoid and papilionoid legumes. In contrast to the symbiosis of legumes with α-proteobacteria, very little is known about the molecular determinants underlying the successful establishment of this mutualistic relationship with β-proteobacteria. In this study, we performed an RNA-sequencing (RNA-seq) analysis of free-living *P. phymatum* growing under nitrogen-replete and -limited conditions, the latter partially mimicking the situation in nitrogen-deprived soils. Among the genes upregulated under nitrogen limitation, we found genes involved in exopolysaccharides production and in motility, two traits relevant for plant root infection. Next, RNA-seq data of *P. phymatum* grown under free-living conditions and from symbiotic root nodules of *Phaseolus vulgaris* (common bean) were generated and compared. Among the genes highly upregulated during symbiosis, we identified—besides the *nif* gene cluster—an operon encoding a potential cytochrome o ubiquinol oxidase (Bphy_3646-49). Bean root nodules induced by a *cyoB* mutant strain showed reduced nitrogenase and nitrogen fixation abilities, suggesting an important role of the cytochrome for respiration inside the nodule. The analysis of mutant strains for the RNA polymerase transcription factor RpoN (σ^54^) and its activator NifA indicated that—similar to the situation in α-rhizobia—*P. phymatum* RpoN and NifA are key regulators during symbiosis with *P. vulgaris*.

## 1. Introduction

Symbiotic nitrogen fixation (SNF) by rhizobia in root nodules of several legumes accounts for a fourth of the N_2_ fixed annually on Earth [1]. Legume-nodulating rhizobia are polyphyletic and include hundreds of species from 14 genera of two bacterial classes: α-proteobacteria (α-rhizobia) and β-proteobacteria (β-rhizobia). The fact that legumes could be nodulated also by β-rhizobia was discovered with the isolation of two *Burkholderia* strains (*Burkholderia tuberum* STM678^T^ and *Burkholderia phymatum* STM815^T^) from the root nodules of the South African legumes *Aspalathus carnosa* and *Cyclopia* spp. [2,3] as well as from *Mimosa* spp. [4,5], and with the isolation of *Cupriavidus taiwanensis* from the nodules of invasive *Mimosa* species in Taiwan [6]. Phylogenetic analyses based on symbiotic genes, such as the nitrogen fixation (*nif*) and nodulation (*nod*) genes, suggested that β-rhizobia have existed as legume symbionts for approximately 50 million years, and that they have evolved separately from α-rhizobia [7,8,9]. Although the South American and Asian β-rhizobia are associated with *Mimosa*, an increasing number of South African β-rhizobia that are associated with diverse papilionoid legumes have been described [3,10,11,12,13,14,15,16]. In addition, naturally occurring symbioses between the papilionoid legumes from the genera *Rhynchosia*, *Dipogon* and *Burkholderia* strains harboring South African-type *nod* genes have been reported [12,17,18]. Phylogenomics approaches led to the proposal to reclassify nodulating and nitrogen-fixing *Burkholderia* species into the new genus *Paraburkholderia* [19,20]. So far, 17 nodulating *Paraburkholderia* species have been described: *P. tuberum*, *P. phymatum* [21], *P. mimosarum* [22], *P. nodosa* [23], *P. sabiae* [24], *P. caribensis* [25], *P. diazotrophica* [26], *P. caballeronis* [27], *P. phenoliruptrix* [25], *P. sprentiae* [28], *P. rhynchosiae* [29], *P. dilworthii* [30], *P. aspalathi* [31], *P. kirstenboschensis* [32], *P. dipogonis* [33], *P. piptadeniae*, and *P. ribeironis* [34]. Currently, only one nodulating and nitrogen-fixing *Burkholderia* strain, *B. symbiotica*, is not a member of the *Paraburkholderia* genus, but instead belongs to the so called “*P. rhizoxinica* group” [35]. 

Nitrogen is the most often limiting nutrient for crop production worldwide, and the *Rhizobium*–legume symbiosis plays a significant role in improving the fertility and productivity of low-N soils. In unfertilized soils, free-living rhizobia developed mechanisms to facilitate N_2_ scavenging from alternative nitrogen sources [36]. The nitrogen regulatory (Ntr) response plays a key role and involves the action of signal transduction PII proteins and of the two-component regulatory system NtrBC [36]. The transcriptional regulator NtrC, together with the alternative σfactor σ^54^ (or RpoN), activates the transcription of genes involved in nitrogen assimilation, such as the glutamine synthetase (*glnA*) and the ammonium transporter (*amtB*). Certain bacteria, such as *Azotobacter*, *Klebsiella*, and *P. phymatum* have been shown to be able to convert atmospheric nitrogen (N_2_) into ammonia in free-living conditions [4,37]. Inside legumes root nodules, rhizobia activate N_2_ fixation and generate ammonia to meet the large needs of the plant. In α-rhizobia, the expression of the symbiotic nitrogen fixation genes (*nif*, coding for the nitrogenase polypeptides, and *fix*, encoding nitrogenase cofactors) is activated by a cascade of signals, which involves low oxygen-sensing regulators such as NifA [38] and FixLJ [39].

For the well-known α-rhizobial symbioses, the different steps leading to a functional root nodule have been characterized in detail [36,40], while for β-rhizobia, very little is known about the molecular mechanisms required to establish and support a symbiosis with their respective host plants. Previous comparative genomics analyses [41,42,43] indicated that, apart from the *nod*–*nif* symbiotic modules, very few genes were specifically shared by the phyletic distinct α- and β-rhizobia, suggesting mechanistic differences between α- and β-rhizobial symbioses. As an example, the *cbb*_3_-type cytochrome oxidase present in all α-rhizobia was not detected in any of the *Paraburkholderia* genomes [44]. Our model system consists of *P. phymatum* STM815^T^ as the rhizobial partner and the agriculturally important legume *Phaseolus vulgaris* (common bean) as the host legume. *P. phymatum* STM815^T^ was first shown to enter symbiosis with *Mimosa* spp. by Elliott and co-workers [4], and many strains have been subsequently isolated from *M. pudica* in both its native and invasive range [4,45,46], strongly suggesting that the original host of STM815^T^ was a *Mimosa* species native to South America [5]. *P. phymatum* STM815^T^ has since been shown to be highly promiscuous, capable of effectively nodulating several *Mimosa* spp. and many other related mimosoid genera [47,48], as well as some legumes in the papilionoid tribe *Phaseoleae* that are normally nodulated by South African *Paraburkholderia* [15,17].

The goal of this study was to gain a better understanding about the genetic basis underlying a β-rhizobial, nitrogen-fixing symbiosis. More specifically, we investigated *P. phymatum* gene expression profile (i) in cells underlying nitrogen starvation, a condition that partially resembles the one that free-living rhizobia encounter in nitrogen-deprived soils before infecting the roots, and (ii) during symbiosis with *P. vulgaris*. 

RNA-sequencing (RNA-seq) experiments showed that *P. phymatum* responded to nitrogen limitation by activating genes involved in the assimilation of nitrogen sources, as well as genes involved in exopolysaccharides (EPS) biosynthesis and motility. These two characteristics were shown in α-rhizobia to be important for the root infection process. The transcriptome of *P. phymatum* inside bean root nodules revealed that—apart from genes involved in nitrogen fixation—other genes potentially important for a symbiotic lifestyle were significantly upregulated in nodules. Mutant strain analyses *in planta* suggested that a cytochrome o ubiquinol oxidase gene cluster (Bphy_3646-49) is important for symbiosis with *P. vulgaris*. Moreover, we show here that two key regulators of nitrogenase expression in α-rhizobia [38], the alternative σ factor RpoN (or σ^54^) and its activator NifA, are equally important for β-rhizobial symbiosis. Additionally, RpoN controls the utilization of nitrate and urea as nitrogen sources, as well as EPS formation.

Finally, this study describes the characterization of three genes important for *P. phymatum – P. vulgaris* symbiosis and provides a rich source of information for the discovery of new functions potentially important for the establishment of the symbiotic interaction between β-rhizobia and papilionoid legumes.

## 2. Materials and Methods

### 2.1. Bacterial Strains, Media, and Cultivation

The bacterial strains, plasmids, and primers employed in this work are listed in Table S1. *Escherichia coli* was grown in Luria-Bertani medium (LB [49]; 10 g of tryptone, 5 g of yeast extract and 4 g NaCl per liter), whereas *P. phymatum* cells were cultivated under aerobic conditions in the modified LB medium without salt. The following antibiotic concentrations were used: chloramphenicol (20 µg/mL for *E. coli* and 80 µg/mL for *P. phymatum*), kanamycin (25 µg/mL for *E. coli* and 50 µg/mL for *P. phymatum*), and nalidixic acid 50 µg/mL for *P. phymatum*. The bacterial cultures for the transcriptomic studies were grown in defined buffered AB minimal medium [50] with 10 mM sodium citrate as the carbon source, supplemented with trace elements [51]. Nitrogen-replete conditions (N) were achieved with 30 mM ammonium chloride (NH_4_Cl), whereas 0.5 mM NH_4_Cl was used to obtain nitrogen-limited (S) conditions. The cultures of *P. phymatum* were grown in 250 mL Erlenmeyer flasks containing 100 mL of medium that were incubated at 30 °C with shaking at 180 rpm for approximately 24 h. The microoxic cultures in AB minimal medium were prepared and grown as previously described [52] in 50 mL of medium in 500 mL rubber-stoppered serum bottles containing 0.5% oxygen (PanGas, Zurich, Switzerland) and 99.5% nitrogen (PanGas, Zurich, Switzerland). The bottles were shaken at 80 rpm; every 9–15 h, the gas phase was exchanged. 

The growth of all *P. phymatum* strains (wild type (wt), *rpoN* mutant, and *rpoN* complemented) was tested with three different nitrogen substrates: ammonium (30 mM), nitrate (30 mM), and urea (15 mM). For each tested strain, the growth of at least two independent cultures was measured.

### 2.2. Plant Growth Conditions and Inoculation

Common bean seedlings (*Phaseolus vulgaris*, cv. Negro jamapa, kindly provided by Professor Eulogio Bedmar, Granada, Spain) were surface-sterilized as previously described [53]. The seeds were deposited on 0.8% agar plates and incubated in the dark at 30 °C. After two days, the germinated seedlings were planted into autoclaved yoghurt jars containing vermiculite (VTT-Group, Muttenz, Switzerland) and 170 mL diluted Jensen medium [54]. The cells were washed twice in AB minimal medium without nitrogen, and 1 mL of the desired bacterial strains (10^5^ bacterial cells) was directly inoculated on the germinated seedling. The plants were grown in the following conditions: the temperature was kept at 22 °C at night and 25 °C during the day; light was supplied for approximately 16 h; humidity was 60%. The root nodules were harvested 21 days post infection (dpi) for analysis.

### 2.3. Determination of Symbiotic Properties

Several symbiotic properties (nodule number, nodule dry weight, nitrogenase activity, and N content) were determined as described previously [55,56,57]. Bean plants inoculated with *P. phymatum* were harvested 21 days post infection (dpi), and the number of nodules on each root was counted. The nodules were dried at 65 °C overnight. The nitrogenase activity was determined by an acetylene reduction assay (ARA). In detail, 1 mL of acetylene (PanGas, Zurich, Switzerland) was injected in a 50 mL tube (Infochroma AG, Zug, Switzerland) containing the root of interest. After co-incubation with the root, 25 µL of the gas from the tube was injected into a gas chromatograph (Agilent, Santa Clara, CA, USA) to analyze the nitrogenase activity, which is represented by the percentage of the ethylene over the acetylene peak [56]. The results were normalized by the dry weight of all the nodules on the plants and the incubation time. At least two independent experiments with five plants per strain were performed. The N content of the shoot was calculated as described before, using the Dumas method (combustion) [57]. Approximately 600 µL of flash-frozen nodules (corresponding roughly to 40 nodules) infected by *P. phymatum* wild type were used for a transcriptome experiment. 

### 2.4. RNA-Sequencing and Data Processing

Total RNA from *P. phymatum* cells grown under free-living nitrogen-replete, nitrogen-limited, or microoxic conditions (see Section 2.1) to the end of the exponential growth phase (optical density at 600 nm of 0.7, 0.4, or 0.5, respectively), and from 600 µL of flash-frozen root nodules was extracted using a modified hot acid phenol protocol [58]. The nodule samples were subjected to an additional acid phenol treatment. Three independent biological replicates were performed per sample. The verification of the complete removal of genomic DNA and a quality check of the RNA were performed as previously described [59]. After DNase treatment, the nodule sample was processed with the Ribo-zero^TM^ Plant-Seed/Root kit (Epicentre, Madison, WI, USA) to remove plant ribosomal RNA (rRNA). After RNA quantification, 100–350 ng were used for cDNA synthesis. Library preparations and purifications were performed using the Encore Complete Prokaryotic RNA-Seq DR Muliplex System (NuGEN, San Carlos, CA, USA), which uses a novel Insert-Dependent Adaptor Cleavage (InDAC) technology for the removal of bacterial rRNA transcripts. Before sequencing, the cDNA libraries were quantified by capillary electrophoresis using the Agilent D1000 Screen Tape System (Agilent, Santa Clara, CA, USA). Illumina single-end sequencing was performed using the HiSeq2500 instrument. Sequence reads were processed and mapped to the *P. phymatum* STM815^T^ genome [48] using CLC Genomics Workbench v7.0 (QIAGEN CLC bio, Aarhus, Denmark), allowing up to two mismatches per read. The unique mapped reads were statistically analyzed for differential expression using the DESeq R-package v1.26 [60]. For DESeq analysis, the top 200 and 500 significantly regulated genes (ranked by ascending *p*-value) were taken into account for nitrogen-limited versus nitrogen-replete (a small regulon was expected), and for symbiotic versus free-living conditions (substantial changes in the expression were expected), respectively. The functional classification of the differentially expressed genes was performed using eggNOG v3.0 [61]. The RNA-seq raw data files are accessible through the GEO Series accession number GSE107381.

### 2.5. Quantitative Reverse Transcription PCR (qRT-PCR) Analysis

The differential expression of *P. phymatum* genes, namely, Bphy_0257 (*amtB*), Bphy_0326 (*rpoN*), Bphy_1479 (*ntrC*), Bphy_1481 (*glnA*), Bphy_3648 (*cyoB*), Bphy_7728 (*nifA*), and Bphy_7753 (*nifH*) was assessed as previously described [59] by quantitative reverse transcription-PCR (qRT-PCR) using the Brilliant III Ultra-Fast SYBR green QPCR master mix (Agilent, Santa Clara, CA, USA) and a Mx3000P instrument (Agilent). Samples of cDNAs were produced as previously described [52]. Three different dilutions (15, 7.5 and 3.75 ng) of each cDNA were used as qRT-PCR templates, and each reaction was done in triplicates. The ΔΔCT method was used to calculate the fold changes in expression [62] using the sigma factor *rpoD* (Bphy_3690) as a housekeeping gene for normalization. For all the primers pairs, an annealing temperature between 55–58 °C was used.

### 2.6. Construction of Paraburkholderia phymatum STM815^T^ Mutant Strains

The genomic DNA (gDNA) of *P. phymatum* was isolated using the DNeasy Blood & Tissue kit (Qiagen, Hilden, Germany), whereas the plasmid DNA from *E. coli* strains was extracted with the QIAprep Spin Miniprep kit (Qiagen). To generate mutant strains, a 300–500 bp-long internal fragment of the genes of interest (*nifA*, *cyoB* and *rpoN*) was amplified with Phusion High-Fidelity DNA polymerase (ThermoFischer, Waltham, MA, USA), using the primers listed in Table S1 (Bphy_x_IM_F and R, where “x” is the gene name). The PCR products of *nifA* and *rpoN* were cloned into pGEM-T Easy (Promega, Madison, WI, USA) and then subcloned into pSHAFT2 as *EcoR*I or *Not*I fragments for *nifA* and *rpoN*. For *cyoB*, the PCR product was digested and directly cloned into the digested pSHAFT2 as *EcoR*I fragment. The resulting plasmids pSHAFT-nifA, pSHAFT-cyoB, and pSHAFT-rpoN were then mobilized into wild-type *P. phymatum* by triparental mating using *E. coli* pRK2013 as a helper strain, generating *P. phymatum nifA* mutant (*nifA* mt, STM815-nifA_Pp_), *cyoB* mutant (*cyoB* mt, STM815-cyoB_Pp_), and *rpoN* mutant (*rpoN* mt, STM815-rpoN_Pp_). The correct genomic integration was verified by PCR using the external oligonucleotides Bphy_nifA_check_F, Bphy_cyoB_check_R, or Bphy_rpoN_check_R in combination with pSHAFTseqFor (Table S1). To construct a *cyoAB* deletional mutant strain, one 452 bp-long fragment (fragment 1), flanking upstream of the gene *cyoA* (Bphy_3649), and one 448 bp-long fragment (fragment 2), flanking downstream of *cyoB* (Bphy_3648), were chosen as recombination sites for deletion mutation. Fragment 1 was amplified with the primers Bphy3649up_XhoI and Bphy3649up_XbaI and fragment 2 was amplified with the primers Bphy3648down_XbaI and Bphy3648down_NotI, using the gDNA of *P. phymatum* as a template. Both fragments were digested with *Xba*I and ligated together. The ligated DNA was amplified by PCR using Bphy3649up_XhoI and Bphy3648down_NotI, then digested by *Xho*I and *Not*I, and subcloned into pSHAFT2. A kanamycin cassette was cut out from pKD4 plasmid using *Xba*I and inserted between the two fragments in pSHAFT2 vector. The resulting plasmid was mobilized into a *P. phymatum* strain, spontaneously resistant to nalidixic acid (NAL, STM815_Nal_), that we generated through spontaneous mutation. In details, 10^8^ *P. phymatum* wild-type cells were plated onto a Tryptone Yeast (TY) agar plate [63] containing nalidixic acid 100 µg/mL, and the survived clones were subsequently purified onto a TY plate containing nalidixic acid 50 µg/mL. The *cyoAB* deletion mutant (Δ*cyoAB*, STM815_Nal_ Δc*yoAB*) was confirmed by PCR using the primers KM_F (annealing to the kanamycin resistance cassette) and Bphy3648_out (annealing to the neighboring gene Bphy_3647). 

To complement the *rpoN* mutant strain, the complete Bphy_0326 open reading frame (ORF) was amplified using the Phusion High-Fidelity DNA polymerase (ThermoFischer), by using the oligonucleotides Bphy_rpoN_c_F/R. The PCR product was digested with *Bam*HI and *Xba*I and cloned into the corresponding sites of pBBR1MSC-2. The complementing plasmid was mobilized into the corresponding *P. phymatum* mutant strain by triparental mating, resulting in the *P. phymatum rpoN* complemented strain (*rpoN* comp). For all the primers pairs, an annealing temperature between 55–58 °C was employed.

### 2.7. Exopolysaccharides Production

Exopolysaccharides production was tested on modified YEM medium plates (1% mannitol, 0.06% yeast extract) as previously described [57,64]. The plates were incubated for 4 days at 30 °C.

### 2.8. Statistical Analysis

The symbiotic properties (nodule number per plant, dry weight per nodule, relative nitrogenase activity, and N content) were analyzed statistically with ANOVA and Tukey’s tests, using the Prism 7 software (version 7.0; La Jolla, CA, USA). For categories distribution, the percentages of upregulated or downregulated genes in each category were calculated by dividing the number of up- and downregulated genes in each category by the total number of genes grouped in the corresponding category. Fischer tests were carried out (online calculator of GraphPad) to assess a possible over- or under-representation of the functional eggNOG categories. 

## 3. Results

### 3.1. Transcript Profiling of P. phymatum STM815^T^ in Response to Nitrogen Limitation

**Figure 1:**
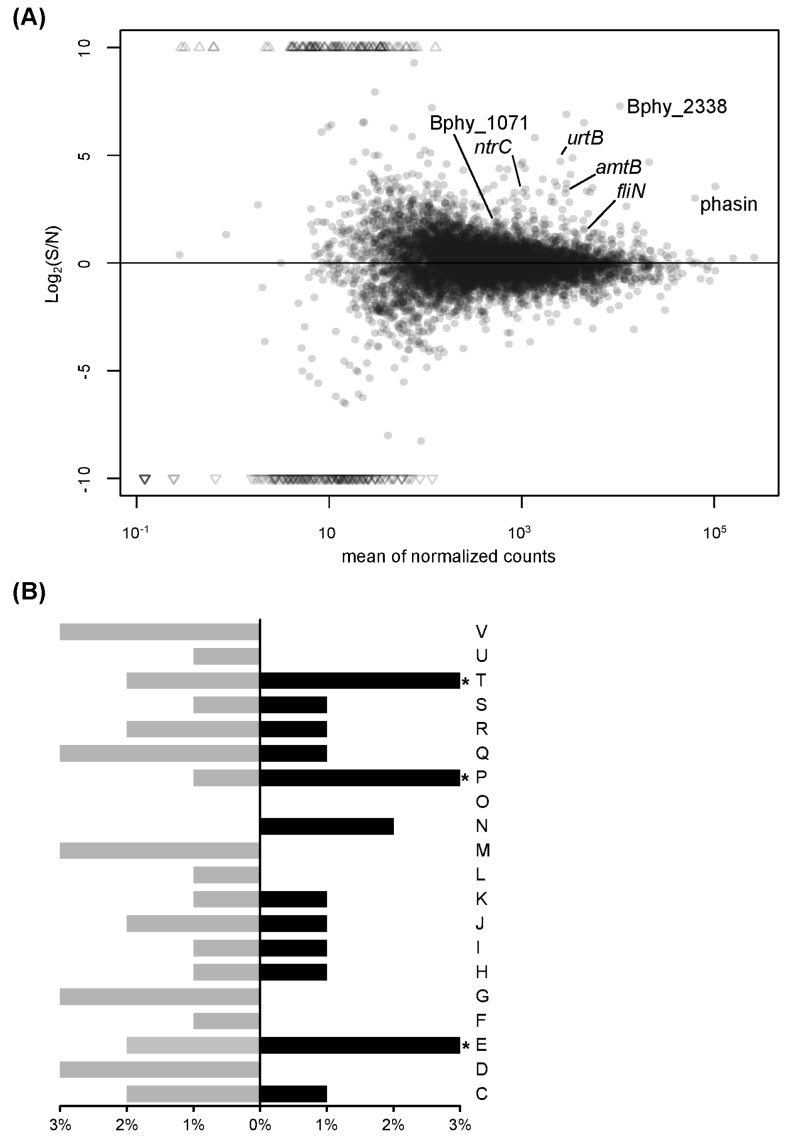
Analysis of the *Paraburkholderia phymatum* transcriptome in response to nitrogen limitation. The MA plot (M log ratios; A averages of normalized counts) displays the logarithm (base 2) of the fold changes in transcripts expression of cells grown under nitrogen-limited (S) versus nitrogen-replete (N) conditions, and the mean of the normalized reads (**A**). Functional categories of the top 200 differentially expressed genes (genes induced in nitrogen starvation are in black, those repressed in grey) according to the eggNOG annotation (**B**). The asterisks (*) indicate statistical significance for over-represented genes in a particular category (*p*-value < 0.01). C, energy production and conversion; E, amino acid transport and metabolism; F, nucleotide transport and metabolism; G, carbohydrate transport and metabolism; H, coenzyme transport and metabolism; I, lipid transport and metabolism; J, translation, ribosomal structure, and biogenesis; K, transcription; L, replication, recombination, and repair; M, cell wall, membrane, and envelope biogenesis; N, cell motility; O, post-translational modification, protein turnover, and chaperon; P, inorganic ion transport and metabolism; Q, secondary metabolites biosynthesis, transport, and catabolism; R, general function prediction only; S, function unknown; T, signal transduction mechanisms; U, intracellular trafficking, secretion, and vesicular transport; V, defense mechanisms.

RNA-seq was employed to first investigate the molecular mechanisms underlying the response to nitrogen limitation in *P. phymatum*, a condition which partially mimics the situation that rhizobia encounter in nitrogen-starved soils. For this, wild-type cells were grown in minimal medium supplemented with ammonium (among the best nitrogen sources used by *P. phymatum* in a Biolog PM3 plate [65]) under nitrogen-replete (N, 30 mM NH_4_Cl) and nitrogen-starved (S, 0.5 mM NH_4_Cl) conditions. From both conditions, three independent biological replicates were processed and sequenced. Reads uniquely mapping to the genome were subjected to a statistical and comparative analysis using DESeq [60]. A graphical visualization of the read intensity (mean of the normalized reads) versus the logarithm (base 2) of the fold change (FC; nitrogen-limited versus nitrogen-replete conditions) are illustrated in Figure 1A. The top 200 differentially expressed genes under nitrogen-limited and -replete conditions are listed in Table S2 (*p*-value ≤ 0.02 and absolute log_2_ (fold change) ≥ 0.87). Out of these, 79 genes showed increased expression when the cells were grown under nitrogen-limiting conditions. 

To explore the functional relevance of the differentially expressed genes, the top 200 nitrogen-regulated genes were assigned to functional categories according to the eggNOG classification system [61]. Certain categories were found to be significantly over-represented: category E (amino acid transport and metabolism), category P (inorganic ion transport and metabolism), and category T (signal transduction mechanisms) (Figure 1B). Among the top differentially expressed genes, several belonged to category E including genes involved in urea transport (Bphy_2251 to Bphy_2254) and *ureA* (Bphy_2258), coding for a urease. Some other genes known to be relevant for nitrogen control in several other bacteria [66,67] were found in category P, such as *amtB* (ammonium transporter, Bphy_0257) and an assimilatory nitrate reductase gene (Bphy_5659-60) with its transporter gene (Bphy_3974-78, excluding Bphy_3975). Finally, a substantial number of transcriptional regulators were classified in category T. These included the nitrogen metabolism transcriptional regulatory gene *ntrC* (Bphy_1479) and the gene coding for a methyl-accepting chemotaxis sensory transducer (Bphy_ 2338), which was the most significantly upregulated gene in nitrogen starvation (Table S2). The gene belonging to a potential EPS cluster (Bphy_1071, the BCAM1005 orthologue in *B. cenocepacia* J2315), as well as the genes coding for a polyhydroxybutyrate (PHB)-associated protein (phasin, Bphy_1467) and for three depolymerases potentially involved in polyhydroxyalkanoates (PHA) and PHB biosynthesis (Bphy_4313, Bphy_4407, and Bphy_5512) also showed significantly enhanced expression under nitrogen-starving conditions. To validate the expression changes observed in the RNA-seq data, several regulated genes important for nitrogen utilization (*amtB*, *ntrC*, *rpoN*, and *glnA*) were subjected to quantitative reverse transcription PCR (Table 1).

### 3.2. Transcript Profiling of P. phymatum STM815^T^ during Symbiosis with P. vulgaris

**Figure 2:**
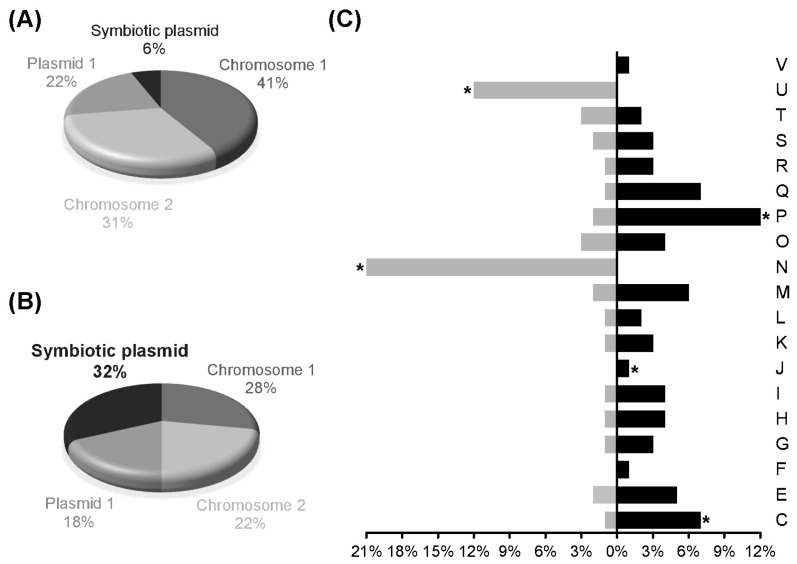
Analysis of the transcripts differentially expressed during symbiosis of *P. phymatum* with *P. vulgaris.* Percentages of the gene distribution among the two chromosomes and two plasmids of the *P. phymatum* genome (**A**), and percentages of the 322 genes significantly induced during symbiosis with respect to their encoding chromosomes or plasmids (**B**). Functional categories of the 500 differentially expressed genes (genes induced in bacteroids shown in black, genes repressed shown in grey) according to the eggNOG annotation (**C**). The 322 genes used for the calculation of the percentages distribution in (B) were obtained from the analysis performed with the R package DESeq (*p*-value ≤ 0.01, log_2_(FC) ≥ 1.6). Asterisks (*) indicate statistical significance for over-represented genes in a particular category (*p*-value < 0.01).

**Figure 3:**
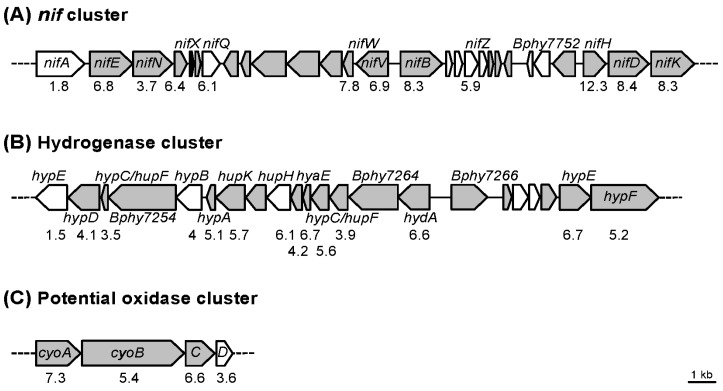
Selected *P. phymatum* gene clusters showing statistically significant upregulation under symbiotic compared to free-living conditions. The following gene clusters are represented: nitrogen fixation (*nif*) (**A**), hydrogenase (**B**), and a potential cytochrome o ubiquinol oxidase cluster (**C**). Gene names are indicated in italic, and the logarithm (base 2) of the fold changes is shown underneath. Genes listed within the top 500 regulated genes (Table S3) are colored in grey.

Next, we investigated the whole transcript profile of *P. phymatum* during symbiosis. For this, bean root nodules induced by *P. phymatum* wild-type were processed 21 days after infection for a RNA-seq analysis. The RNA from *P. phymatum* cells grown in minimal medium under aerobic conditions was used to provide a gene expression baseline for comparison. Among three independent biological replicates per condition, we obtained—per nodule sample—between 4.2 and 5.3 million reads uniquely mapping to the *P. phymatum* genome. The transcript profiles of *P. phymatum* grown under symbiotic and free-living conditions were compared by DESeq analysis. Among the top 500 differentially expressed genes (DESeq analysis *p*-value ≤ 0.01 and absolute log_2_ (Fold change) ≥ 1.6) (Table S3), 322 were significantly upregulated. Out of these 322, 32% are located on the symbiotic plasmid (0.59 Mb), 28% on chromosome 1 (3.48 Mb), 22% on chromosome 2 (2.7 Mb), and 18% on plasmid 1 (1.9 Mb) (Figure 2B). This indicated a strong enrichment of regulated genes on the symbiotic plasmid that otherwise harbors only 6% of the genes of the whole genome (Figure 2A). Furthermore, the top 500 differentially regulated genes were assigned to functional categories (Figure 2C). Among these, 178 showed significant decreased expression in nodules. As displayed in Figure 2C, the eggNOG category N (cell motility) comprised 21% of the genes downregulated inside the nodule. A couple of them were involved in flagellar biosynthesis (Bphy_2940-41, *fliOP*; Bphy_2962, *flgF*). In addition, category U (intracellular trafficking, secretion, and vesicular transport), which contains a partial cluster for a type IV secretion system (Bphy_7525 to Bphy_7536), was significantly over-represented among the genes with reduced expression inside the nodule. Analysis of the 322 genes significantly upregulated in nodules, indicated that category C (energy production and conversion) was over-represented, among which were the genes known to be important for symbiotic functions such as nitrogen fixation (*nif* cluster) and hydrogenase (Figure 3A,B). We also found a gene coding for an isocitrate lyase (Bphy_1368) and a potential cytochrome o ubiquinol oxidase cluster (Bphy_3646-49), which may play a role for respiration inside the nodule (Figure 3C). In the over-represented functional category P (inorganic ion transport and metabolism) we identified several ATP-binding cassette (ABC)-transporters for sulfate (Bphy_1627 and Bphy_1629), nitrate/sulphonate/bicarbonate (Bphy_3603), phosphate (Bphy_3120 and Bphy_4622), aliphatic sulphonate (Bphy_5226), taurine (Bphy_6080), and urea (Bphy_2251-52). In addition, two interesting genes displayed increased expression in nodules: Bphy_1467, coding for a phasin-like protein potentially involved in PHB stability, and a PHB depolymerase (Bphy_4407). In order to mimic the transition towards conditions of extremely low oxygen inside the nodules, we additionally grew *P. phymatum* under microoxic conditions (supplemented with 0.5% oxygen) and performed a transcriptome analysis [68]. As expected, under microoxic conditions the genes coding for the nitrogenase were highly expressed and upregulated (Table 2). The cytochrome o ubiquinol oxidase cluster (Bphy_3646-49) that was upregulated in symbiosis also showed increased expression under reduced oxygen availability (Table 2). The upregulated genes belonging to several of the over-represented categories discussed above are listed in Table 3. 

We observed an overlap of 38 genes with increased expression in both nitrogen-limited growth conditions (Table S2) and during symbiosis with bean (Table S3). Among those, we found two genes involved in PHB production: *phaP* (Bphy_1467), encoding a phasin regulating PHB stability by binding to PHB-granula [69], and a PHB depolymerase (Bphy_4407). The regulatory gene *ntrC*, which is part of the two-component regulatory system (2CRS) NtrB/NtrC, known to be important for nitrogen control in other organisms [64], also showed significantly higher expression under nitrogen-limited conditions and during symbiosis.

A complete list with all *P. phymatum* genes and logarithm (base 2) of the fold changes in expression in nitrogen-starved versus -replete conditions, as well as during symbiosis is shown in Table S4.

### 3.3. Role of nifA, rpoN, and cyoB Genes during Symbiosis

The transcription of the *nif* genes in α-rhizobia is regulated by the key regulator NifA [38,70], which works together with the σ factor RpoN (σ^54^). Remarkably, *P. phymatum* lacks the typical high-oxygen-affinity *cbb*_3_-type cytochrome oxidase present in α-rhizobia [44], but a potential cytochrome o ubiquinol oxidase cluster (Bphy_3646-49) was found among the significantly and highly upregulated genes inside the nodule (Table 3). To investigate the role of the *nifA*, *rpoN*, and *cyoB* genes during symbiosis in our β-rhizobial model system, *P. phymatum nifA* (Bphy_7728), *rpoN* (Bphy_0326), and *cyoB* (Bphy_3648) mutant strains were constructed, as well as a Δ*cyoAB* deletion mutant. The deletion mutant was generated using a strain resistant to nalidixic acid (NAL) as a baseline (see Section 2.6).

**Figure 4:**
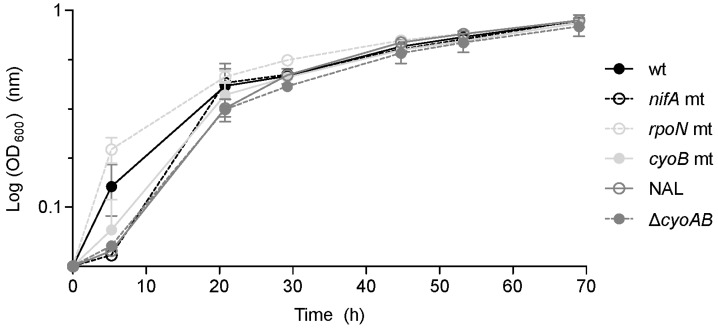
Growth of *P. phymatum* wild-type (wt), nalidixic acid-resistant wild-type (NAL), and mutant (mt) strains (*nifA*, *rpoN*, *cyoB*, and Δ*cyoAB*) under microoxic conditions (see Section 2.1). Whiskers indicate standard deviation (SD), *n* ≥ 2. OD_600_, Optical density at λ = 600 nm.

First, the growth of the four mutant strains (*nifA*, *rpoN*, *cyoB*, and Δ*cyoAB*), as well as of the wild type (wt) and of the nalidixic acid-resistant wild-type (NAL) was monitored in microaerobic conditions (0.5% O_2_), i.e., in conditions that mimic those inside the nodules. In general, a slight growth delay of the mutant strains (*nifA*, *cyoB*, and Δ*cyoAB*) compared to the wild-type was observed. On the contrary, the *rpoN* mutant strain displayed faster growth in the exponential phase, but finally reached a similar final optical density at λ = 600 nm (OD_600_) (Figure 4).

**Figure 5:**
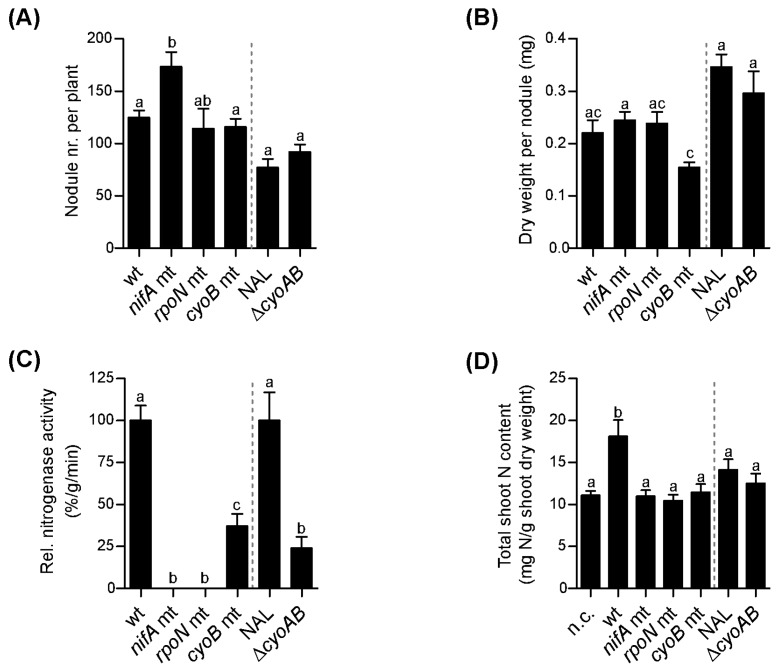
Comparison of the symbiotic properties of *P. vulgaris* (bean) plants inoculated with different *P. phymatum* wild-type and mutant strains: wild-type (wt) and the respective *nifA*, *rpoN*, *cyoB* mutants, as well as a nalidixic acid-resistant wild-type strain (NAL) and the respective Δ*cyoAB* mutant. Number of nodules per plant (**A**), dry weight per nodule (**B**), relative nitrogenase activity (**C**), and nitrogen content (**D**) were quantified 21 dpi. Here, the combined results of at least two independent experiments are shown. Error bars indicate the standard error of the mean (SEM). For each histogram, values with the same letter are not significantly different (as assessed with ANOVA, Tukey’s test with *p*-value ≤ 0.05). Histograms after the grey dashed line were analyzed by an unpaired student *t*-test (*p*-value ≤ 0.05); the values with the same letter are not statistically significant.

The relevance of these genes during symbiotic life was then tested using our host legume *P. vulgaris* (bean). The same amount of colony forming units (CFU; 10^5^) of wild-type and mutant strain cells was inoculated on bean seedlings. After 3 weeks of growth in the green house, several key symbiotic parameters of the nodules, such as their number, the dry weight, their relative nitrogenase activity, as well as their N content were determined. At 21 days post infection (dpi), the roots inoculated with the *nifA* mutant strain had significantly more nodules compared to bean plants inoculated with the other strains (Figure 5A). No significant difference in the overall nodule number was observed for the other mutant strains compared to their respective wild type. However, the dry weight per nodule of plants infected by the *nifA* mutant strain was comparable to that of plants inoculated with either the wild type or the *rpoN* mutant strains (Figure 5B). In contrast to the nodules induced by the wild-type strain, those induced by the *nifA* and *rpoN* mutant strains showed no nitrogenase activity (Figure 5C). The nodules induced by the *cyoB* and the Δ*cyoAB* mutants showed a 65% and 75% reduction in the relative nitrogenase activity compared to their wild-type strain, respectively. In line with the results for the nitrogenase activity, plants infected with a *nifA* or an *rpoN* mutant had an N-content comparable with that of uninfected bean plants. This was significantly lower than the N-content measured in plants containing *P. phymatum* wild type (Figure 5D). Plants infected by the *cyoB* mutant and the Δ*cyoAB* strains displayed an N-content that was similar to the one of uninoculated plants. Together, these results suggest that, besides *nifA* and *rpoN*, also *cyoB* plays an important role for an efficient establishment of a symbiosis with bean.

### 3.4. Role of RpoN in Free-Living Conditions

It is known from several bacteria that RpoN (σ^54^) is involved in the assimilation of nitrogen sources [59,71,72]. To investigate if σ^54^ has a similar role in *P. phymatum*, we examined the ability of the wild-type, the *rpoN* mutant, and the complemented *rpoN* strains to grow in minimal medium in the presence of different nitrogen sources. We found that the *rpoN* mutant strain was unable to utilize nitrate (NO_3_^−^) and urea (CH_4_N_2_O) as sole nitrogen sources, and that this defect could be restored in the *rpoN* complemented strain (Table 4). As expected, the *nifA* mutant strain was not impaired in the utilization of the tested nitrogen sources. Taken together, these results suggested that RpoN is involved in the regulation of nitrite and urea assimilation in *P. phymatum*. 

**Figure 6:**
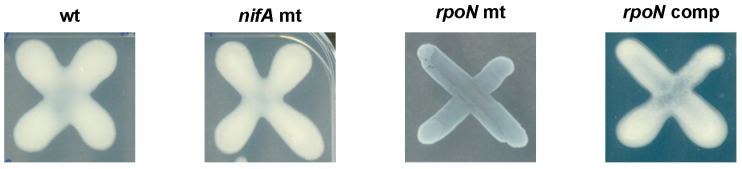
Exopolysaccharide (EPS) production of the *nifA* mutant, the *rpoN* mutant, and the complemented strains tested on plates supplemented with 0.06% of yeast extract. Plates were incubated for four days. At least three independent replicates were performed per strain.

Since EPS production has been shown to be dependent on the availability of nitrogen sources and on the presence of RpoN [59,73], EPS production by the wild-type, the *rpoN* mutant, and the complemented strains, as well as by the *nifA strain* (as control), was examined on Yeast Extract Mannitol medium (YEM) plates containing 1% mannitol and 0.06% yeast extract. While the *rpoN* mutant strain (Figure 6) displayed a reduced amount of EPS compared to the wild type, the *nifA* mutant and the *rpoN* complemented strain produced wild-type levels of EPS. Interestingly, when YEM medium was supplemented with 0.6% of yeast extract, there was nearly no visible difference between the *rpoN* mutant strain and the wild-type strain in EPS production (data not shown), suggesting that *rpoN* controls EPS production under nitrogen starvation.

## 4. Discussion

Members of the β-proteobacterial have been discovered in 2001 to be able to enter a nitrogen-fixing symbiosis with legumes [4]. In contrast to the α-rhizobial symbiosis with host legumes, where the different steps leading to the establishment of a successful symbiosis are well studied, very little is known about the molecular mechanisms that are relevant for these steps in β-rhizobial symbioses. Here, we used an RNA-sequencing approach to provide a comprehensive view on the gene expression profile of *P. phymatum* grown in either normal or nitrogen-limited free-living conditions, and as bacteroids in symbiosis with *P. vulgaris*. To the best of our knowledge, this is the first transcriptome study from root nodules formed by a β-rhizobial strain. By testing the gene expression profile in nitrogen-limited conditions, we aimed to partially mimic the conditions that rhizobia encounter in soils lacking nitrogen and in our laboratory settings before colonizing the roots of legumes. Apart from genes involved in nitrogen metabolism, a gene cluster potentially involved in EPS biosynthesis (Bhy_1056-Bphy_1077) showed increased expression when nitrogen became limited. Exopolysaccharides were shown in several rhizobia to be required for root hairs attachment [74] and infection [75], the first two steps of the cascade. The gene that showed the most significant and the second highest upregulation during nitrogen starvation encodes for a potential methyl-accepting chemotaxis sensory transducer (Bphy_2338). The presence of another gene involved in motility among the top regulated genes (the flagellar gene *fliL*) suggests that the cells react to a nitrogen-limited environment by changing their movement behavior, which is a crucial trait for the successful colonization and infection of host legume roots [76]. The future construction and characterization of mutant strains will shed light on the importance of EPS and flagella in a nitrogen-starved environment. The expression of *nod* genes, encoding the *P. phymatum* Nod factor required for the recognition of the symbiotic partner, changed only slightly in response to nitrogen starvation (Figure S1). The expression of genes in the *nif* cluster did not significantly change when the cells were grown under nitrogen limitation, suggesting that—similar to the situation in α-rhizobia—the presence of a reduced amount of nitrogen is not sufficient to activate the expression of the *nif* cluster. In contrast, a low-oxygen environment highly induced the expression of the genes coding for the nitrogenase (Table 2). Among the 38 genes commonly upregulated, we found *ntrC*, which codes for a transcriptional regulators known to be important for nitrogen control (Ntr) in other organisms [64]. While the Ntr system is usually switched off during nitrogen fixation in symbiotic α-rhizobia [36], in free-living diazotrophs such as *Azospirillum brasilense* the two-component regulatory system NtrB/NtrC has been shown to be involved in the regulation of nitrogenase activity [77]. Two genes coding for a urea ABC transporter (Bphy_2251-52) were also among the genes commonly induced under nitrogen-starving and symbiotic conditions. This may suggest that this organic compound serves an important role in free-living and symbiotic metabolism.

As expected, the expression of the *nif* gene cluster (including genes from Bphy_7728 to Bphy_7755, Figure 3A), was found significantly upregulated inside nodules induced by *P. phymatum*. Unlike most α-rhizobia [78], *P. phymatum* contains a *nifV* homolog in the genome (upstream of *nifB*), which is also highly induced during symbiosis. This gene encodes a homocitrate synthase that synthesizes homocitrate—a component of the Fe–Mo cofactor of the nitrogenase—which has been shown to be important in diazotrophs to reduce N_2_ in free-living conditions [79,80,81]. The presence of *nifV* in *P. phymatum* may explain the ability of this bacterium to fix nitrogen in free-living conditions [4]. Among the upregulated genes in symbiosis, we found a potential four-component oxidase cluster also annotated as a cytochrome o ubiquinol oxidase complex. This cluster (Bphy_3646-49, *cyoABCD*) was also significantly upregulated in a preliminary transcriptome analysis performed on free-living cells grown in microaerobic conditions compared to cells growing aerobically, suggesting that this heme–copper respiratory oxidase could be used by *P. phymatum* to respire inside root nodules. Indeed, the classical *cbb*_3_-oxidase crucial for symbiosis in α-rhizobia was detected neither in the *P. phymatum* genome nor in other symbiotic *Paraburkholderia* species [44]. The construction of a *cyoB* insertion mutant and a *cyoAB* deletion mutant provided proof that this cluster is indeed important for an efficient symbiotic interaction. Bean plants inoculated with these mutants showed a significantly reduced nitrogenase activity and a lower N content compared to plants infected with the wild type. In previous studies [82,83], three genes were shown to be important during symbiosis with *M. pudica*: Bphy_0456, involved in the biosynthesis of branched-chain amino acids, Bphy_0685, coding for a fructose 1,6-bisphosphatase, and Bphy_0266 (*gpmA*), coding for a phosphoglycerate mutase. The expression of these three genes was not regulated in bean root nodules compared to free-living conditions, suggesting that *P. phymatum* may upregulate a different set of genes in its natural host plant *Mimosa*. Interestingly, and in contrast to the situation in α-rhizobia [84,85], a gene coding for an isocitrate lyase (Bphy_1368) was found inside the top 500 regulated genes in *P. phymatum* bacteroids, suggesting that the glyoxylate shunt pathway is active during β-rhizobial symbiosis. In previous studies on *Bradyrhizobium* sp. ORS278, the *ccbL1* gene, which codes for a ribulose 1,5 bis-phosphate carboxylase oxygenase (RuBisCO) needed for carbon fixation, was proven to have a critical role in symbiotic nitrogen fixation [86]. We noticed here that the *P. phymatum* gene coding for ribulose-1,5-bisphosphate carboxylase/oxygenase (RuBisCO; Bphy_6497, 77% amino acid identity to *ccbL1* or BRADO1659 of *Bradyrhizobium* sp. ORS278) was also upregulated inside root nodules, suggesting a possible role of this enzyme also in β-rhizobial symbiosis. The mutation of two *P. phymatum* regulatory genes known to play a key role for α-rhizobial symbiosis [38]—the alternative sigma factor RpoN and its activator NifA—showed that both regulators are also important for the regulation of nitrogenase activity (Figure 5C). Nodules infected by a *nifA* mutant strain were impaired in nitrogen fixation, even 28 dpi [87]. An increased number of nodules in grape-like structures were produced in plants infected with the *P. phymatum nifA* mutant strain. A similar phenotype was observed in the nodules of another legume, soybean, that were induced by a *Bradyrhizobium diazoefficiens nifA* mutant [88]. Using a metabolomics approach on *Bradyrhizobium diazoefficiens* nodules, we previously speculated that such a phenotype could be due to a defense reaction of the legume evoked by the *nifA* mutant, involving an increased production of phytoalexins [89]. Since NifA is an activator protein of the alternative sigma factor σ^54^ (RpoN), we constructed an *rpoN* mutant, which indeed did not show any nitrogenase activity. In addition, the *rpoN* gene was found highly expressed in all conditions tested, i.e., nitrogen-replete and -limited conditions and during symbiosis, suggesting that RpoN may play an important role not only in symbiosis but also in free-living conditions. In fact, the utilization of nitrogen sources as well as the EPS production were affected in this strain. In a closely related *Burkholderia* strain belonging to the pathogenic clade, RpoN was shown to play a role in free-living conditions and also in vivo, where a mutant showed reduced virulence in the *Caenorhabditis elegans* infection model [59]. 

In summary, this first analysis of bacterial gene expression in symbiotic bean root nodules induced by a β-rhizobial strain revealed new insights into this recently discovered symbiosis. It provides a rich basis for a further dissection of the molecular mechanisms underlying this symbiotic association and for the elucidation of the mechanistic differences between β-rhizobial and the much better characterized α-rhizobial symbioses.

## Figures and Tables

**Table 1:** Quantitative reverse transcription PCR (qRT-PCR) and RNA-sequencing (RNA-seq) analysis of genes with induced expression under nitrogen-starved conditions.

Locus ID ^1^	Description ^1^	Gene Name	FC (S vs. N) ^2^	FC (S vs. N) ^3^
Bphy_0257	Ammonium transporter	*amtB*	19.7 ± 4.6	10.9
Bphy_0326	RNA polymerase factor σ^54^	*rpoN*	1.4 ± 0.1	0.7
Bphy_1479	Nitrogen metabolism transcriptional regulator	*ntrC*	8.8 ± 1.2	9.7
Bphy_1481	Glutamine synthetase, type I	*glnA*	4.0 ± 0.8	1.9

^1^ Locus identifier and description is given according to the GenBank files (NC_010622.1, NC_010623.1, NC_010625.1, NC_010627.1); ^2^ fold change (FC) in qRT-PCR expression by comparing the wild-type strain grown under nitrogen-limited (S) and nitrogen-replete conditions (N); ^3^ fold change (FC) determined by DESeq analysis comparing the transcriptome profile of cells grown under nitrogen-limited (S) and nitrogen-replete conditions (N).

**Table 2:** qRT-PCR and RNA-seq analysis of genes with induced expression during symbiosis and microoxic conditions.

Locus ID ^1^	Description ^1^	Gene Name	FC (Bacteroids vs. Free-Living) ^2^	FC (MO vs. O) ^3^
Bphy_0326	RNA polymerase factor σ^54^	*rpoN*	2.1 ± 0.5	1.6
Bphy_1479	Nitrogen metabolism transcriptional regulator	*ntrC*	6.6 ± 1.6	0.9
Bphy_3648	Cytochrome o ubiquinol oxidase, subunit I	*cyoB*	201.8 ± 44.0	68.3
Bphy_7728	Transcriptional regulator	*nifA*	19.1 ± 2.4	4.2
Bphy_7753	Nitrogenase reductase	*nifH*	898.4 ± 174.2	567.0

^1^ Locus identifier and description is given according to the GenBank files (NC_010622.1, NC_010623.1, NC_010625.1, NC_010627.1); ^2^ fold change (FC) in qRT-PCR expression for the wild-type strain grown in symbiotic conditions (bacteroids) compared to free-living conditions; ^3^ fold change (FC) determined by DESeq analysis comparing the transcriptome profile of cells grown in microoxic conditions (MO) with that of cells grown under oxic conditions (O).

**Table 3:** List of 68 genes showing increased expression in bacteroids compared to free-living conditions and belonging to an over-represented eggNOG category (Fischer test *p*-value < 0.01).

Locus ID ^1^	Description ^1^	Gene Name	Log_2_FC (Bacteroids vs. Free-Living) ^2^
*Energy production and conversion*			
Bphy_1368	isocitrate lyase		3.1
Bphy_1649	alkanesulfonate monooxygenase		6.0
Bphy_1848	2-oxoacid dehydrogenase subunit E1		2.9
Bphy_2272	FAD linked oxidase domain-containing protein		2.9
Bphy_3647	cytochrome o ubiquinol oxidase, subunit III	*cyoC*	6.6
Bphy_3648	cytochrome o ubiquinol oxidase, subunit I	*cyoB*	5.4
Bphy_3649	ubiquinol oxidase, subunit II	*cyoA*	7.3
Bphy_3685	phosphate acetyltransferase		2.0
Bphy_4116	rubrerythrin		1.9
Bphy_4949	aldehyde dehydrogenase		2.6
Bphy_5235	alkanesulfonate monooxygenase		6.2
Bphy_5817	putative flavodoxin		3.6
Bphy_6055	hypothetical protein		INF
Bphy_6505	formylmethanofuran dehydrogenase subunit A		1.9
Bphy_6506	formylmethanofuran-tetrahydromethanopterin formyltransferase		3.9
Bphy_6671	2Fe-2S iron-sulfur cluster binding domain-containing protein		INF
Bphy_6672	carbon-monoxide dehydrogenase (acceptor)		3.7
Bphy_6673	aldehyde oxidase and xanthine dehydrogenase molybdopterin binding		4.5
Bphy_7231	cytochrome ce class I		2.8
Bphy_7232	xenobiotic (desulfurization)monooxygenase subunit A		5.3
Bphy_7262	hydrogenase expression/formation protein		5.6
Bphy_7263	Ni/Fe-hydrogenase, b-type cytochrome subunit		3.9
Bphy_7264	nickel-dependent hydrogenase large subunit		6.4
Bphy_7265	hydrogenase (NiFe) small subunit	*hydA*	6.6
Bphy_7406	aldehyde dehydrogenase		7.3
Bphy_7729	nitrogenase MoFe cofactor biosynthesis protein	*nifE*	6.8
Bphy_7730	nitrogenase molybdenum-cofactor biosynthesis protein	*nifN*	3.7
Bphy_7733	ferredoxin III, *nif*-specific		6.3
Bphy_7737	electron-transferring-flavoprotein dehydrogenase	*fixC*	6.8
Bphy_7738	electron transfer flavoprotein α/β-subunit	*fixB*	7.1
Bphy_7739	electron transfer flavoprotein α/β-subunit	*fixA*	7.8
Bphy_7754	nitrogenase molybdenum-iron protein α chain	*nifD*	8.4
Bphy_7755	nitrogenase molybdenum-iron protein β chain	*nifK*	8.3
Bphy_7804	electron transfer flavoprotein α/β-subunit		3.9
*Inorganic ion transport and metabolism*			
Bphy_0882	phosphate ABC transporter, periplasmic protein		4.3
Bphy_0883	phosphate transporter permease subunit	*pstC*	2.5
Bphy_0885	phosphate transporter ATP-binding protein		2.6
Bphy_1627	sulfate ABC transporter inner membrane subunit	*cysW*	2.2
Bphy_1629	sulfate ABC transporter, periplasmic protein		5.2
Bphy_1647	ABC transporter-like protein		5.9
Bphy_1648	binding-protein-dependent transport systems		3.3
Bphy_2231	sulfate adenylyltransferase large subunit		2.3
Bphy_2521	catalase		4.9
Bphy_3120	phosphate ABC transporter, periplasmic protein		4.2
Bphy_3602	ABC transporter related		4.0
Bphy_3603	nitrate/sulfonate/bicarbonate ABC transporter, periplasmic protein		2.6
Bphy_3854	phosphate transporter		2.7
Bphy_4233	Rieske (2Fe-2S) domain-containing protein		2.9
Bphy_4622	phosphonate ABC transporter binding protein		6.7
Bphy_5040	lipoprotein		7.8
Bphy_5065	2-aminoethylphosphonate ABC transporter, 2-aminoethylphosphonate binding protein		2.0
Bphy_5226	aliphatic sulfonate ABC transporter, periplasmic protein		4.0
Bphy_5227	substrate-binding region of ABC-type glycine betaine transport system		9.0
Bphy_5229	aliphatic sulfonate ABC transporter, periplasmic protein		5.8
Bphy_5232	rhodanese domain-containing protein		5.6
Bphy_6080	taurine ABC transporter, periplasmic binding protein		7.5
Bphy_6081	ABC transporter related		4.2
Bphy_6550	metallophosphoesterase		3.5
Bphy_7233	ABC transporter related		3.7
Bphy_7234	binding-protein-dependent transport systems		4.6
Bphy_7235	binding-protein-dependent transport systems		4.8
Bphy_7236	ABC sulfate ester transporter, periplasmic protein		4.3
Bphy_7645	binding-protein-dependent transport systems		3.2
Bphy_7646	binding-protein-dependent transport systems		4.4
Bphy_7647	ABC transporter related		4.9
Bphy_7753	nitrogenase reductase	*nifH*	12.3
Bphy_7808	nitrogenase reductase	*nifH*	INF
*Translation, ribosomal structure and biogenesis*			
Bphy_2864	GCN5-like *N*-acetyltransferase		4.2

^1^ Locus identifier and description is given according to the GenBank files (NC_010622.1, NC_010623.1, NC_010625.1, NC_010627.1); ^2^ logarithm (base 2) of the fold change (FC) in expression for the wild-type strain grown in symbiotic conditions (bacteroids) compared with free-living conditions: INF, not computable because the read number for the wild type grown under free-living conditions was 0; FAD, Flavin Adenine Dinucleotide; ABC, ATP-Binding Cassette; ATP, Adenosine TriPhospate.

**Table 4:** Utilization of various nitrogen sources by *P. phymatum* wild type, *nifA*, *rpoN* mutant, and *rpoN* complemented strains ^1^.

Nitrogen Source (s)		Utilization of Nitrogen		
	wt	*nifA* mt	*rpoN* mt	*rpoN* complemented
30 mM NH_4_Cl	+	+	+	+
0.5 mM NH_4_Cl	+	+	+	±
30 mM NO_3_^−^	+	+	-	+
15 mM CH_4_N_2_O	+	+	-	+

^1^ Growth was assessed with at least two independent replicates by measuring the optical density at 600 nm after incubation in ABC minimal medium supplemented with several nitrogen sources for 30 h at 30 °C and 220 rpm. The “+” sign corresponds to OD_600_ ≥ 0.6 for ammonium, ≥ 0.3 for nitrate, and ≥ 0.6 for urea; the “±” sign corresponds to OD_600_ ≥ 0.4 for ammonium.

## Supplementary Materials

The following are available online at www.mdpi.com/2073-4425/8/12/389/s1. Figure S1: *P. phymatum* nodulation (*nod*) gene cluster. Gene names are indicated in italic, whereas the logarithm (base 2) of the fold changes in transcript expression of the genes under nitrogen-limiting conditions are shown underneath. The upregulated genes (log_2_FC ≥ 0.5) are colored in grey, Table S1: bacterial strains, plasmids, and oligonucleotides used in this study; Table S2: list of the top 200 differentially regulated genes in nitrogen-starved (S) versus nitrogen-repleted (N) conditions (DESeq analysis; *p*-value ≤ 0.02, absolute log_2_[FC] ≥ 0.87); Table S3: list of the top 500 differentially regulated genes in bacteroids compared to free-living conditions (DESeq analysis; *p*-value ≤ 0.01 and absolute log_2_(Fold change) ≥ 1.6); Table S4: list of all *P. phymatum* genes with the logarithm (base 2) of the fold changes in expression, and the *p*-values in nitrogen-starved (S) versus -replete (N) conditions, as well as during symbiosis versus free-living conditions, as calculated by DESeq analysis.
