# Supplementary material for: Transcriptome Analysis of Paraburkholderia phymatum under Nitrogen Starvation and during Symbiosis with Phaseolus Vulgaris
Source: Genes (Basel). 2017 Dec 15;8(12):389. doi: 10.3390/genes8120389 (PMC5748707; doi:10.3390/genes8120389)
Supplement: Supplementary file 1 [file genes-08-00389-s001.zip › Supplementary material/Table S1.docx]

# **Table S1:** Bacterial strains, plasmids and oligonucleotides used in this study.

| Strain or plasmid | Description | Reference |
| --- | --- | --- |
| Strains |  |  |
| *E. coli* |  |  |
| cc118λ-pir | Δ(ara-leu) *araD* Δ*lacX74 galE galK phoA20 thi1 rpsE rpoB argE(Am) recAl* λ pir; Strep^R^ | [1] |
| Top10 | Δ*lacX74 ara* Δ*139*Δ(*ara-leu*) | Invitrogen |
|  |  |  |
| *P. phymatum* |  |  |
| STM815 | Wild type | [2] |
| STM815_Nal_ | Wild type spontaneously resistant to nalidixic acid; Nal^R^ | This study |
| STM815-nifA_Pp_ | *nifA*_Pp_::pSHAFT2 mutant of STM815; Cm^R^ | This study |
| STM815-cyoB_Pp_ | *cyoB*_Pp_::pSHAFT2 mutant of STM815; Cm^R^ | This study |
| STM815-rpoN_Pp_ | *rpoN*_Pp_::pSHAFT2 mutant of STM815; Cm^R^ | This study |
| STM815_Nal_ Δ*cyoAB* | Δ*cyoAB* of STM815_Nal_; Nal^R^, Km^R^ | This study |
|  |  |  |
| Plasmids |  |  |
| pGEM-T Easy | Cloning vector; Amp^R^, *lac*Z | Promega |
| pRK2013 | Helper plasmid; Km^R^ | [3] |
| pSHAFT2 | Suicide plasmid; Cm^R^ | [4] |
| pBBR1MCS-2 | Broad host-range cloning vector; Km^R^ | [5] |
| pSHAFT-*nifA* | pSHAFT2 containing a 414 bp internal fragment of Bphy_7728 for mutagenesis; Cm^R^ | This study |
| pSHAFT-*cyoB* | pSHAFT2 containing a 332 bp internal fragment of Bphy_3648 for mutagenesis; Cm^R^ | This study |
| pSHAFT-*rpoN* | pSHAFT2 containing a 489 bp internal fragment of Bphy_0326 for mutagenesis; Cm^R^ | This study |
| pBBR2-*rpoN* | pBBR1MCS-2 containing Bphy_0326 for complementation; Km^R^ | This study |
|  |  |  |
| Oligonucleotides | **Sequence^1^** | **Source** |
| pSHAFTseqFor | CTTCAGCTGATGTGTGATAACATACT | K. Agnoli unpublished |
| Bphy_nifA_IM_F | ACGGACATCTGTGCAGTTTG | This study |
| Bphy_nifA_IM_R | CACGAATGGTTTCCTTACGC | This study |
| Bphy_nifA_check_F | CGGAGCTCAAATCCATGC | This study |
| Bphy_cyoB_IM_F | AAAAAGAATTCCGATCACAAGCGAATTGG | This study |
| Bphy_cyoB_IM_R | AAAAAAAGCTTCGATCACCAGCGAAATGT | This study |
| Bphy_cyoB _check_R | CATCTTCATCAGCGACATGC | This study |
| Bphy_rpoN _IM_F | CTACGCTCGAACTGCAACAG | This study |
| Bphy_rpoN_IM_R | CCAGTATTTCGTCGAACGTG | This study |
| Bphy_rpoN_check_R | GGATCCTTGACAGGCTACTGGCAGGT | This study |
| Bphy_rpoN_c_F | TTTGGATCCATGAAAGCCAGCCTCCAA | This study |
| Bphy_rpoN_c_R | TTTTCTAGACTACAGAGACTTGCGCAG | This study |
| Bphy3649up_XhoI | AAAAAACTCGAGGCCAAAAGACAGAAGACGA | This study |
| Bphy3649up_XbaI | AAAAAATCTAGAAGTTGACATCCCGCAAGAC | This study |
| Bphy3648down_XbaI | AAAAAATCTAGAGTGGTACGGACGATCGAAAA | This study |
| Bphy3648down_NotI | AAAAAAGCGGCCGCAGGTGCGAGAACTCGTTGAG | This study |
| Bphy3648_out | ACGAAGAACGACGACAGGAA | This study |
| KM_R | TTTCTACGTGTTCCGCTTCC | This study |
| Bphy3941_F | AGATCGTCAGCGAGAACCAT | This study |
| Bphy3941_R | TCTGACGGTTGGTTTCCTTC | This study |
| Bphy7728_F | ATCTGGAACGAATGGTGAGG | This study |
| Bphy7728_R | GGTTGAAACGATCGAGGAAA | This study |
| Bphy1479_F | ATCTGCTGGAATCCGAACTG | This study |
| Bphy1479_R | GCGATTCGAGATTCTGATGTG | This study |
| Bphy7808_F | GGCGTGGACTATGTGTCGTA | This study |
| Bphy7808_R | GATGCCCTTCGAGATGTTGT | This study |
| Bphy3648_F | GCGAATTGGGGTGATGTA | This study |
| Bphy3648_R | TGAACAGACCCACCATCA | This study |
| Bphy0257_F | GCTCGTCACAGTGATCTGGA | This study |
| Bphy0257_R | ACTTCATCCGGTCAGCAAAC | This study |
| Bphy0326_F | CTACGCTCGAACTGCAACAG | This study |
| Bphy0326_R | AACCCAGCCCGTTGTACTC | This study |
| Bphy1481_F | GTCAAGATCGGTTCGGAAGA | This study |
| Bphy1481_R | CTGACCTTGACCTGCCACTT | This study |

^1^restriction sites are underlined.

**References**

1. Herrero, M.; de Lorenzo, V.; Timmis, K. N. Transposon vectors containing non-antibiotic resistance selection markers for cloning and stable chromosomal insertion of foreign genes in gram-negative bacteria. *J Bacteriol* **1990**, *172*, 6557–6567.

2. Moulin, L.; Munive, A.; Dreyfus, B.; Boivin-Masson, C. Nodulation of legumes by members of the beta-subclass of Proteobacteria. *Nature* **2001**, *411*, 948–950, doi:10.1038/35082070.

3. Phadnis, S. H.; Berg, D. E. Identification of base pairs in the outside end of insertion sequence IS50 that are needed for IS50 and Tn5 transposition. *Proc Natl Acad Sci U S A* **1987**, *84*, 9118–9122.

4. Shastri, S.; Spiewak, H. L.; Sofoluwe, A.; Eidsvaag, V. A.; Asghar, A. H.; Pereira, T.; Bull, E. H.; Butt, A. T.; Thomas, M. S. An efficient system for the generation of marked genetic mutants in members of the genus *Burkholderia*. *Plasmid* **2017**, *89*, 49–56, doi:10.1016/j.plasmid.2016.11.002.

5. Kovach, M. E.; Elzer, P. H.; Steven Hill, D.; Robertson, G. T.; Farris, M. a.; Roop, R. M.; Peterson, K. M. Four new derivatives of the broad-host-range cloning vector pBBR1MCS, carrying different antibiotic-resistance cassettes. *Gene* **1995**, *166*, 175–176, doi:10.1016/0378-1119(95)00584-1.
