# Supplementary figures and images for: Transcriptome Analysis of Paraburkholderia phymatum under Nitrogen Starvation and during Symbiosis with Phaseolus Vulgaris
Source: Genes (Basel). 2017 Dec 15;8(12):389. doi: 10.3390/genes8120389 (PMC5748707; doi:10.3390/genes8120389)

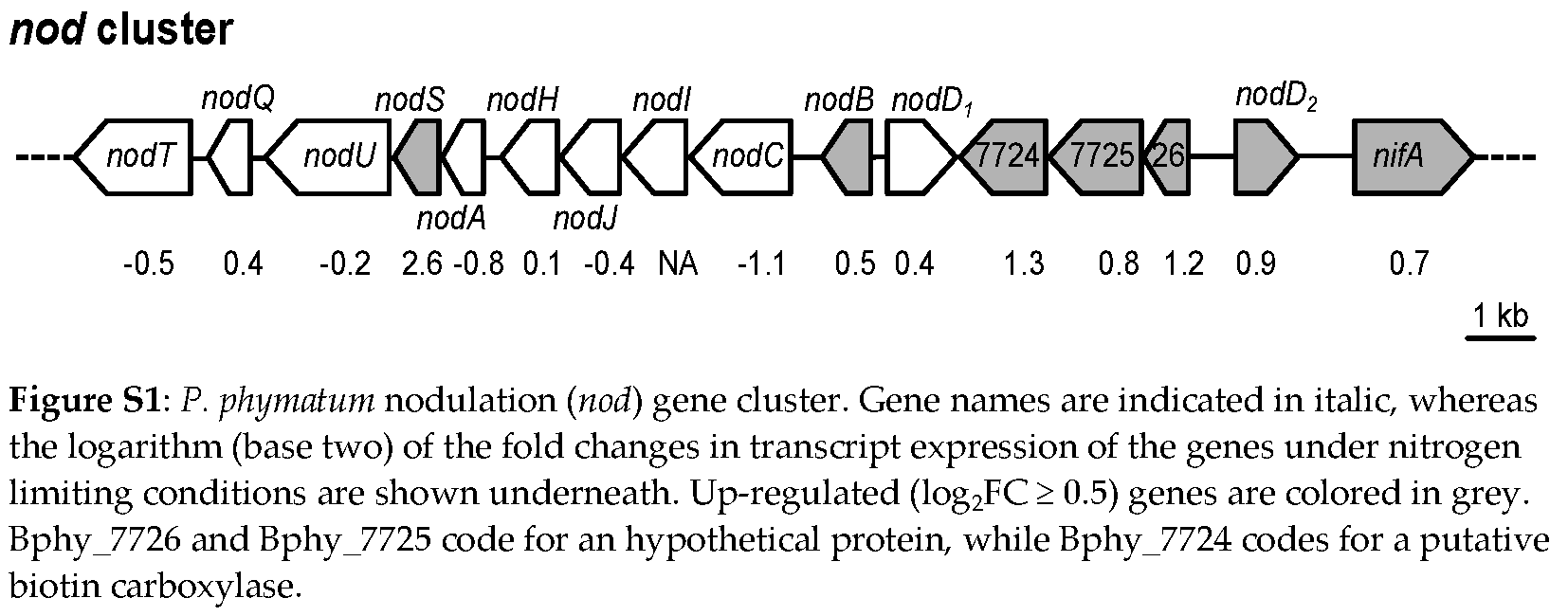

Supplement: Supplementary file 1 [file genes-08-00389-s001.zip › Supplementary material/Figure_S1.tiff]
